# BCL-2 family protein expression and platinum drug resistance in ovarian carcinoma

**DOI:** 10.1054/bjoc.1999.0939

**Published:** 2000-01-18

**Authors:** P J Beale, P Rogers, F Boxall, S Y Sharp, L R Kelland

**Affiliations:** CRC Centre for Cancer Therapeutics, Institute of Cancer Research, 15 Cotswold Rd, Belmont, Sutton, SM2 5NG, UK

**Keywords:** platinum, BCL-2, ovarian carcinoma, resistance

## Abstract

The expression of the BCL-2 family proteins, BCL-2, BAX, BCL_XL_and BAK have been determined in a panel of 12 human ovarian carcinoma cell lines encompassing a wide range in sensitivity to cisplatin. Whereas BAX, BCL_XL_and BAK levels did not correlate with sensitivity, there was a statistically significant inverse correlation (*r* = –0.81;*P* = 0.002) between growth inhibition by cisplatin and BCL-2 levels. In sublines possessing acquired resistance to various platinum-based drugs or across a panel of human ovarian carcinoma xenografts, there was no consistent pattern of BCL-2 expression. Two relatively sensitive lines (A2780 and CH1) have been stably transfected with bcl-2 and bcl_XL_respectively and two relatively resistant lines (A2780cisR and SKOV-3) stably transfected with bax. Overexpression of BCL-2 in A2780 cells led to resistance to cisplatin compared to the vector control when assayed at 48 h post-drug incubation but a significant increase in sensitivity at 96 h. Relative rates of apoptosis at 48- and 96-h post-cisplatin exposure mirrored the growth inhibition. There was no significant difference in sensitivity of the pair of lines by clonogenic assay. No significant changes in chemosensitivity to a variety of DNA-damaging or tubulin-interactive agents were observed in the remaining transfected lines. Taken together, these results suggest that, in human ovarian carcinoma cells, high BCL-2 levels (either naturally occurring or through gene transfection) confers a trend towards sensitivity not resistance to platinum drugs. © 2000 Cancer Research Campaign


*British Journal of Cancer* (2000) **82**(2), 436–440
© 2000 Cancer Research Campaign
Article no. bjoc.1999.0939

### BCL-2 family protein expression and platinum drug resistance in ovarian carcinoma

**PJ Beale, P Rogers, F Boxall, SY Sharp and LR Kelland**

CRC Centre for Cancer Therapeutics, Institute of Cancer Research, 15 Cotswold Rd, Belmont, Sutton SM2 5NG, UK

**Summary** The expression of the BCL-2 family proteins, BCL-2, BAX, $BCL_{XL}$ and BAK have been determined in a panel of 12 human ovarian carcinoma cell lines encompassing a wide range in sensitivity to cisplatin. Whereas BAX, $BCL_{XL}$ and BAK levels did not correlate with sensitivity, there was a statistically significant inverse correlation ($r = -0.81$; $P = 0.002$) between growth inhibition by cisplatin and BCL-2 levels. In sublines possessing acquired resistance to various platinum-based drugs or across a panel of human ovarian carcinoma xenografts, there was no consistent pattern of BCL-2 expression. Two relatively sensitive lines (A2780 and CH1) have been stably transfected with bcl-2 and $bcl_{XL}$ respectively and two relatively resistant lines (A2780cisR and SKOV-3) stably transfected with bax. Overexpression of BCL-2 in A2780 cells led to resistance to cisplatin compared to the vector control when assayed at 48 h post-drug incubation but a significant increase in sensitivity at 96 h. Relative rates of apoptosis at 48- and 96-h post-cisplatin exposure mirrored the growth inhibition. There was no significant difference in sensitivity of the pair of lines by clonogenic assay. No significant changes in chemosensitivity to a variety of DNA-damaging or tubulin-interactive agents were observed in the remaining transfected lines. Taken together, these results suggest that, in human ovarian carcinoma cells, high BCL-2 levels (either naturally occurring or through gene transfection) confers a trend towards sensitivity not resistance to platinum drugs. © 2000 Cancer Research Campaign

**Keywords**: platinum; BCL-2; ovarian carcinoma; resistance

In recent years, it has been proposed that the suppression of apoptosis following a cytotoxic insult may represent a key determinant of poor chemotherapeutic response (Dive and Hickman, 1991). Foremost among the proteins thought to be involved is the BCL-2 family of pro-apoptotic (e.g. BAX, BCL-Xs, BAK, BAD, BIK, BID) or anti-apoptotic (e.g. BCL-2, BCL-$X_L$, MCL-1, BCL-W) members (reviewed in Reed, 1997).

As part of our platinum drug discovery programme, we have established panels of human ovarian carcinoma cell lines, sensitive and resistant to cisplatin and analogues (Kelland et al, 1992*a*; Holford et al, 1998). Furthermore, for many of the cell lines, xenograft counterparts have been established (Kelland et al, 1992*b*). The aim of this study was to determine whether the levels of proteins of the BCL-2 family may correlate with the platinum drug sensitivity and resistance of these ovarian cell lines and xenografts. In addition, as a further test of the role of Bcl-2 family proteins in determining ovarian cancer cell line platinum drug sensitivity, one sensitive cell line (A2780) has been transfected with Bcl-2 and one (CH1) with $Bclx_L$. Furthermore, two resistant lines (SKOV3 and A2780cisR) have been transfected with bax and chemosensitivity patterns to both DNA-interactive and tubulin-interactive anticancer drugs determined.

### MATERIALS AND METHODS

### Cell lines

A panel of parental human ovarian carcinoma cell lines were used in this study – (A2780, CH1, 41M, LK1, LK2, PXN94, PA1, OVCAR3, OAW42, SKOV3, 59M and OV1P) and lines made resistant to cisplatin and other platinum analogues (A2780cisR, ZD0473R, JM149R, JM335R; CH1cisR, JM216R, ZD0473R, JM118R, JM149R, JM221R, JM335R; 41 McisR, JM216R, JM221R; OVCAR3 carboR; OV1PcisR, PXN94tetraR). Parent and platinum drug resistant lines have been previously described (Kelland et al, 1992*a*, 1992*b*; Holford et al, 1998).

Transfections were performed using lipofectamine (Life Technologies). Full-length cDNAs and/or vectors for bcl-2, bax and $bclX_L$ were kindly supplied by Professor D Kerr (Birmingham, UK), Dr S Korsmeyer (St Louis, MO, USA) and Dr G Nunez (Ann Arbor, MI, USA) respectively. A2780 was stably transfected with full-length bcl-2 cDNA using SV-40 as the promoter, and selection by resistance to neomycin. A2780cisR and SKOV-3 lines were transfected with a construct of full-length human bax coupled to an HA tag cloned into the bicistronic plasmid vector pIRES-P (EMBL:Z75185) under the control of the cytomegalovirus promoter, or with an empty vector as control, using selection with puromycin. CH1 is a relatively cisplatin sensitive cell line and was transfected with Bclxl using the bicistronic vector pIRES-P or an empty vector and selected for resistance to puromycin. In all cases, expression of the transfected gene and resulting protein was confirmed by immunoblotting (see below).

All cell lines were grown as monolayers in Dulbecco's modified Eagle medium (DMEM) supplemented with 10% fetal calf serum (FCS; Imperial Laboratories, Andover, UK), 2 mM L-glutamine and 0.5 μg $ml^{-1}$ hydrocortisone in a humidified 6% carbon dioxide, 94% air atmosphere.

Human ovarian xenografts models used in the study were CH1, CH1 cisR, PXN87, PXN871 (Iproplatin resistant), PXN95, PXN951, PXN65, PXN100, SKOV3 and HX62 (Kelland et al, 1992*b*; Jones et al, 1993). These were grown in the flanks of nude mice and routinely passaged on after reaching around 12 mm in size.

*Received 26 July 1999*
*Accepted 14 September 1999*

*Correspondence to:* LR Kelland

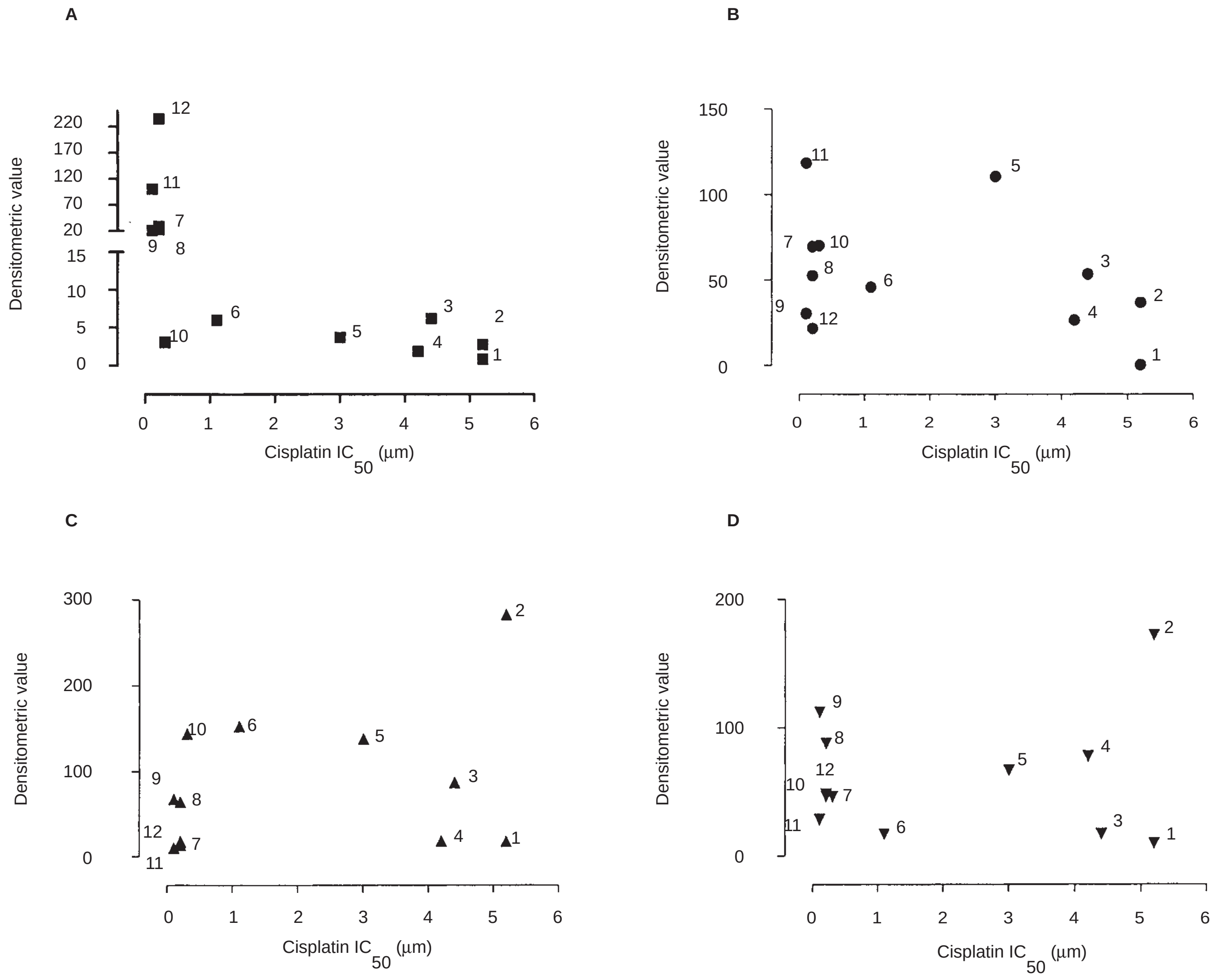

**Figure 1** Relationships between sensitivity to cisplatin and protein levels of (**A**) BCL-2, (**B**) BAX, (**C**) $BCL_{XL}$ and (**D**) BAK for 12 human ovarian carcinoma cell lines (1 = OVCAR-3, 2 = 59 M, 3 = SKOV-3, 4 = OVIP, 5 = PXN94, 6 = OAW42, 7 = PA1, 8 = LK1, 9 = LK2, 10 = A2780, 11 = CH1 and 12 = 41 M)

### Chemotherapy agents and other chemicals

Platinum drugs were synthesized by Johnson Matthey Technology Centre (Reading, Berkshire, UK) or AnorMED (Langley, BC, Canada). Structures of platinum agents have been published previously (Kelland et al, 1992*a*, 1992*b*; Holford et al, 1998). Doxorubicin, etoposide and paclitaxel were obtained through the Royal Marsden NHS Trust Hospital Pharmacy. All other chemicals were obtained from Sigma Chemicals (Poole, UK) unless otherwise stated.

### Cytotoxicity assays

Sulphorhodamine B (SRB) and microtetrazolium (MTT) growth inhibition assays were performed to assess chemosensitivity as described previously (Kelland et al, 1992*a*). Unless otherwise indicated, drug exposure was continuous for 96 h. For clonogenic survival assays, 1000 cells of A2780BCL-2 and A2780 vector (neo) cell lines were allowed to attach overnight in triplicate T25 flasks. Cisplatin exposure was for 2 h. After 7 days, colonies with >50 cells were stained with methylene blue and counted.

### Rate of apoptosis

This was determined by measuring cellular detachment from the monolayer following drug exposure as described previously (Ormerod et al, 1996).

### Western blotting

This was performed as described previously using sodium dodecyl sulphate polyacrylamide gel electrophoresis (SDS-PAGE) separation and transfer to nitrocellulose membranes and visualization by enhanced chemiluminescence reagents (ECL) (Holford et al, 1998). Semiquantitative levels were determined by scanning densitometry and analysis by Imagequant software. Densitometric levels were adjusted to a control level from one line for the ovarian cell lines and another line for the xenografts. Antibodies used for detection were BCL-2 (DAKO), BAX (Santa Cruz N-20), $BCL_{XL}$ (Santa Cruz S-18), BAK (Calbiochem-AB-1), p21 (Santa-Cruz C-19) and p53 (Santa Cruz DO-1).

© 2000 Cancer Research Campaign

**Table 1** BCL-2 family protein levels in a panel of 12 human ovarian carcinoma xenografts of differing responsiveness to platinum drugs

| | BCL-2 protein (units) | BAX protein (units) | BCL-$_{XL}$ protein (units) | BAK protein (units) | Growth delay[b] (days) | Tumour volume doubling time[c] (days) |
|---|---|---|---|---|---|---|
| PXN/65 | 11.5 | 80 | 118.6 | 75.1 | >238 | 19.0 |
| PXN/100 | 3.0 | 100 | 142.6 | 85 | >259 | 11.5 |
| PXN87 | ND | 55.9 | ND | 150 | 180 | 12.1 |
| PXN87I[a] | ND | 12.3 | ND | 135.3 | 15.0 | 12 |
| CH1 | 35.8 | 90.8 | 154.9 | 60.4 | 29.2 | 4.7 |
| CH1cisR | 44.4 | 176.4 | 115.1 | 88.8 | 10.4 | 3.6 |
| HX110 | 98.2 | 44.5 | 47.9 | 229.3 | 41.7 | 3.8 |
| HX110P[+] | 59.7 | 9.8 | 28.5 | 80.7 | 6.5 | 8.0 |
| PXN95 | ND | 19.1 | ND | 371.4 | 81 | 17.7 |
| PXN95I[+] | ND | 12.7 | ND | 193.4 | 11.5 | 8.5 |
| HX62 | ND | 50 | 70.2 | 104.2 | 0.5 | 4.5 |
| SKOV-3 | 4.9 | 55 | 86.6 | 185.4 | 10.7 | 6.2 |

[a]P = carboplatin resistant; I = iproplatin resistant. [b,c]Growth delay to cisplatin (weekly intraperitoneal dosing for 4 weeks; 4–8 mg $kg^{-1}$) and tumour doubling time values taken from Kelland et al (1992a, b) and Jones et al (1993).

### Statistical analyses

When appropriate, statistical significance was tested using a two-tailed Student's *t*-test; a *P*-value of ≤0.05 was considered as significant. All values shown are means with the corresponding standard error of the mean. Correlation analyses were determined by linear regression or by Spearman rank and performed using GraphPad Prism Software.

### RESULTS

### Characterization of BCL-2 family protein levels in human ovarian cell lines and xenografts

Levels of BCL-2, BAX, BCLxL and BAK in our panel of 12 parental human ovarian cell lines as measured by Western blotting and subsequent densitometry are presented in Figure 1A–D respectively and related to sensitivity to cisplatin. The densitometric levels may be divided into three bands; < 10 equals absent or very low levels; 10–60 represents low to moderate levels; and levels above 60 represent moderate to high levels. Levels of BCL-2 appeared inversely related to cisplatin sensitivity; many of the resistant cell lines such as SKOV-3, 59M, OVCAR-3 possessed relatively low levels while high levels were observed in the cisplatin sensitive lines 41M and CH1. Spearman rank correlation analysis revealed a significant inverse relationship between sensitivity and BCL-2 levels ($r$ = –0.81; $P$ = 0.0022). There was no significant relationship between the level of any of the remaining proteins and the 96-h $IC_{50}$ μM to cisplatin in the cell lines (BAX $r$ = –0.32; $BCL_{XL}$ $r$ = 0.47; BAK $r$ = –0.17).

BCL-2 protein levels in acquired platinum drug-resistant cell lines showed no consistent pattern (data not shown). For example, in the CH1 (and OVCAR-3) cell lines, platinum- and platinum analogue-resistant cell lines had higher levels of BCL-2 protein (1.1- to 2.2-fold), but in the 41M (and OV1P and PXN94) lines, the platinum-resistant lines had significantly lower levels (0.22–0.49). Interestingly, in the A2780 cell lines BCL-2 was undetectable in the parent and cisR lines, but in the A2780/ZD0473 R line the levels were significantly higher.

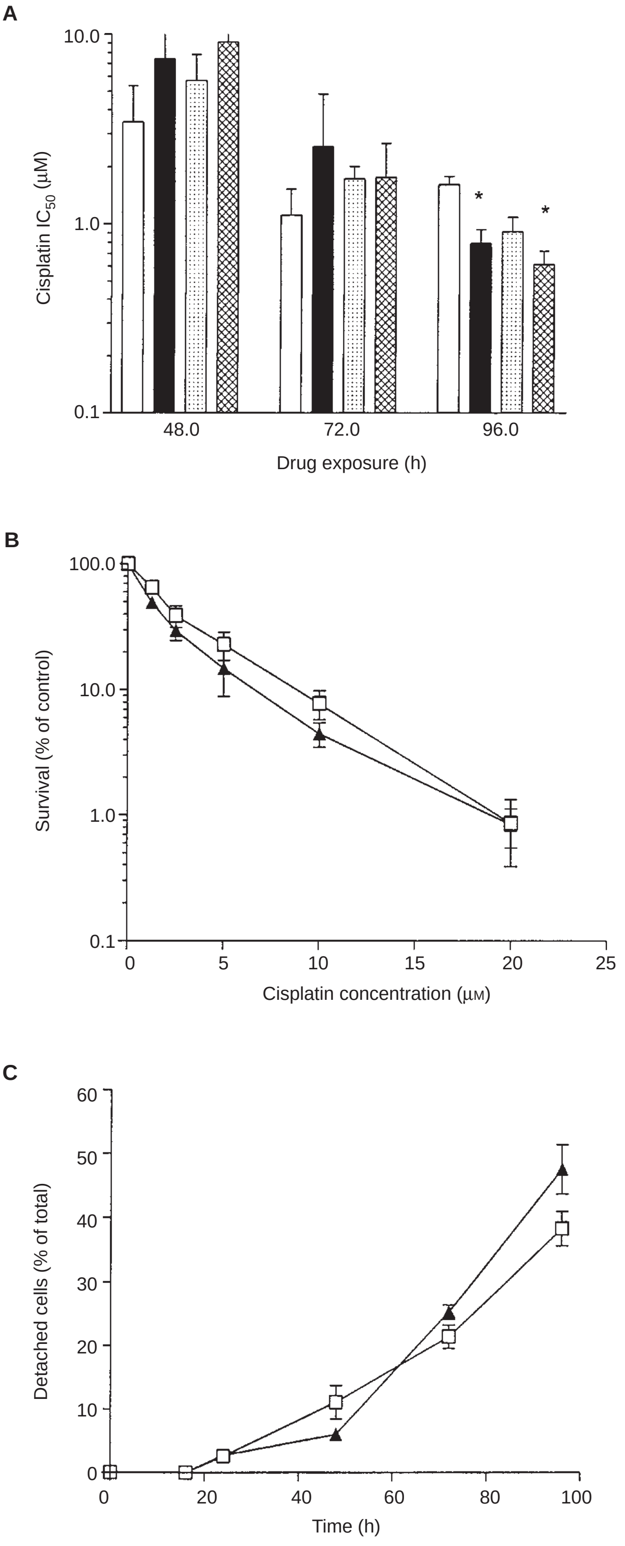

**Figure 2** Sensitivity of A2780 vector (neo) control and A2780BCL-2 transfect to cisplatin by (**A**) MTT (vector control = open bars; A2780BCL2 transfect solid bars) or SRB assay (vector control = dotted bars; A2780BCL2 transfect cross-hatched bars) at 48, 72 or 96 h post-drug exposure or (**B**) clonogenic cell survival assay following 2 h exposure to cisplatin for A2780 vector (neo) control (□) or A2780BCL-2 transfect (▲) or (C) induction of apoptosis as measured by cellular detachment following 2 h exposure to cisplatin for A2780 vector (neo) control (□) or A2780BCL-2 transfect (▲). * denotes statistical significance ($P$ < 0.05)

 © 2000 Cancer Research Campaign

Levels of the BCL-2 family proteins in a panel of human ovarian carcinoma xenografts of varying responsivenesss to cisplatin are shown in Table 1. Spearman correlation analysis showed no significant correlation ($P > 0.05$) between responsiveness to cisplatin (growth delay) and levels of BCL2, BAX, $BCL_{XL}$ or BAK. As with the pairs of in vitro cell lines, there was no consistent pattern in protein levels.

### Characterization of an A2780 BCL-2 stable transfected line

The A2780BCL-2 line, in contrast to the vector control, was shown to have enhanced expression of the BCL-2 protein (data not shown), similar doubling times (21.1 ± 0.9 h and 20.1 ± 1.1 h respectively) and similar levels of platinum accumulation and DNA platination following exposure to cisplatin (data not shown). The sensitivity of the A2780 vector (neo) control and BCL-2 transfect to cisplatin was determined by 3 independent assays (MTT, SRB – Figure 2A; and clonogenic – Figure 2B) and, for the short-term colourimetric assays at 48-, 72- and 96-h post-drug exposure. Figure 2A reveals an interesting pattern of relative response vs time of assay. Whereas at 48 and 72 h post-drug exposure the BCL-2-transfected line was more resistant to cisplatin than the vector control, at 96 h the reverse was true with the BCL-2 line being significantly more sensitive than the vector control by both assays. Results of a colony forming assay (Figure 2B) showed no significant difference in sensitivity between the lines. Results using the 96 h SRB growth inhibition assay for a range of platinum and other standard antitcancer agents of diverse mechanisms of action (e.g. topoisomerase I and II inhibitors, tubulin interactive drugs) showed a tendency in all cases for the A2780BCL-2 transfect to be more sensitive than the vector control although this only reached statistical significance in the cases of cisplatin and the topoisomerase I inhibitor camptothecin (data not shown).

The rate of cellular detachment following exposure to 10 μM cisplatin for 2 h was used an indicator of apoptosis in these cell lines (Ormerod et al, 1996). Results are shown in Figure 2C. At 48 h post-exposure, there was greater detachment with the vector control line (11.1 ± 2.6% vs 6.0 ± 0.41% in A2780BCL-2). However, by 96 h, the trend was in the opposite direction (38.3 ± 2.7% in vector control vs 47.5 ± 3.8% in A2780BCL-2).

Following exposure to 25 μM cisplatin (5 times the $IC_{50}$) for 2 h, in the A2780BCL-2 transfect there was induction of BCL-2, BAX and BAK, some induction of p53 and p21 but no marked induction of $BCL_{XL}$. In contrast, the most notable differences for the vector control were: (1) a decrease in the level of BAK, (2) no induction of BCL-2, and (3) greater induction of p53 and p21. The responses of the BAX and $BCL_{XL}$ proteins were similar in both lines (data not shown).

### Characterization of A2780cisR and SKOV-3 bax transfects and CH1 $BCL_{XL}$ transfect

BAX protein levels were 1.4-fold higher in A2780cisR and sixfold higher in SKOV3 transfects when compared to vector controls. The sensitivity of the transfected lines as measured by the 96-h SRB cytotoxicity assay to four DNA-interactive agents (cisplatin, ZD0473, doxorubicin and etoposide) and four tubulin-interactive agents (paclitaxel, docetaxel, vinblastine and colchicine) showed that there were no significant differences in sensitivity within the two cell line pairs to any of the drugs evaluated.

$BCL_{XL}$ levels were fourfold higher in the CH1-transfected line compared with the vector control. The 96-h SRB cytotoxicity assay results showed a significant increase in resistance in the transfected line compared with the control for ZD0473 alone ($IC_{50}$ in μM of 0.6 ± 0.08 for CH1puro and 1.53 ± 0.4 for CH1$BCL_{XL}$; RF 2.56, $P = 0.05$), although the parental non-transfected CH1 line showed a similar sensitivity to that of the $BCL_{XL}$ transfect. For all of the remaining drugs there was no significant difference in sensitivity between the two isogenic $BCL_{XL}$ lines (e.g. cisplatin $IC_{50}$ in μM of 0.201 ± 0.04 for CH1puro and 0.26 ± 0.02 for CH1$BCL_{XL}$; RF 1.3) (remaining data not shown).

### DISCUSSION

This study has addressed the possible role of BCL-2 and three other commonly described members, two pro-apoptotic (BAX and BAK) and one other anti-apoptotic ($BCL_{XL}$) in determining the cisplatin sensitivity of panels of human ovarian carcinoma cell lines and xenografts. The major findings from this study were: (1) overexpression of BCL-2 protein in human ovarian carcinoma cell lines, either naturally or via stable gene transfection, tended to confer *sensitivity* to cisplatin rather than resistance. A statistically significant inverse correlation ($r$ of – 0.81) was observed between BCL-2 levels and cisplatin sensitivity in 12 parental ovarian cell lines and transfection of Bcl-2 into A2780 cells conferred a significant increase in sensitivity to cisplatin (and the topoisomerase I inhibitor camptothecin) when using a 96 h growth inhibition assay; (2) no significant correlations were obtained when comparing in vitro drug sensitivity to levels of BAX, $BCL_{XL}$ or BAK or when comparing levels of all four proteins to the platinum response of 12 ovarian cancer xenografts; (3) introduction of Bax into relatively cisplatin resistant lines or $BCL_{XL}$ into a relatively sensitive line did not confer marked changes in chemosensitivity.

Our in vitro results with BCL2 in ovarian cancer differ to many other in vitro studies, especially using cells of haematopoietic origin. Herein, overexpression has been shown to promote cell survival by inhibiting apoptosis induced by a variety of stimuli including chemotherapeutic agents (reviewed in Reed, 1997). However, two other independent studies also using human epithelial cells (HeLa) and exposure to either aphidicolin or etoposide have shown an inhibition of apoptosis through overexpression of bcl-2 but no difference in clonogenic cell survival (Yin and Schimke, 1995; Lock and Stribinskiene, 1996). Hence these data agree more closely with our findings where we observed less apoptosis and low level resistance conferred by overexpression of BCL-2 in A2780 cells at 48 h post-treatment, the opposite at 96 h and no difference in clonogenic cell survival. Interestingly, with respect to ovarian cancer, transfection of bcl-2 into A2780 cells caused low level (2.1- to 3.5-fold) cisplatin resistance but in this study, growth inhibition was assessed (by MTT assay) at only 48 h post-cisplatin treatment (Eliopoulos et al, 1995; Herod et al, 1996). Results from longer term or colony forming assays were not reported. Clinically, in lymphoma, neuroblastoma and leukaemia the overexpression of BCL-2 is associated with an inferior prognosis (reviewed in Reed, 1997). However, in breast, non-

© 2000 Cancer Research Campaign 

small-cell lung, renal cell, colon and ovarian carcinoma higher expression of BCL-2 is associated with a better outcome (e.g. Herod et al, 1996).

There was no consistent pattern of BCL-2 expression across various acquired platinum drug-resistant lines, the most notable change being increased expression in a subline possessing resistance to ZD0473 (AMD473), a sterically hindered platinum drug now in phase I trial (Holford et al, 1998). However, it is unclear to what extent, if any, the BCL-2 overexpression contributes to the acquired resistant phenotype in this line. We observed a differential induction of BAK following cisplatin exposure in the A2780 vector control versus BCL-2 transfect with marked induction in the presence of BCL-2 overexpression compared to a reduction in levels of this pro-apoptotic protein in the vector control. Thus, up-regulation of BAK may contribute to the enhanced sensitivity of the BCL-2-transfected line to cisplatin observed by 4-day growth inhibition assays. Also when using A2780 cells, an up-regulation of BAK (and BAX) was observed following paclitaxel or cisplatin treatment (Jones et al, 1998).

Overall, our results suggest that, at least in human ovarian carcinoma cell lines, high BCL-2 levels (either naturally occurring or through gene transfection) confers a trend towards sensitivity not resistance to platinum drugs.

### ACKNOWLEDGEMENTS

Studies were supported by grants from the Cancer Research Campaign (UK). We thank Dr S Hobbs for guidance on vector construction and M Valenti and L Brunton for growth of human tumour xenografts.

### REFERENCES

Dive C and Hickman JA (1991) Drug–target interactions: only the first step in the commitment to a programmed cell death? *Br J Cancer* **64**: 192–196

Eliopoulos A, Kerr D, Herod J, Hodgkins, Krajewski S, Reed J and Young L (1995) The control of apoptosis and drug resistance in ovarian cancer: influence of p53 and Bcl-2. *Oncogene* **11**: 1217–1228

Herod JJO, Eliopoulos AG, Warwick J, Niedobitek G, Young LS and Kerr DJ (1996) The prognostic significance of Bcl-2 and p53 expression in ovarian carcinoma. *Cancer Res* **56**: 2178–2184

Holford J, Sharp SY, Murrer BA, Abrams M and Kelland LR (1998) In vitro circumvention of cisplatin-resistance by the novel sterically hindered platinum complex AMD473. *Br J Cancer* **77**: 366–373

Jones M, Siracky J, Kelland LR and Harrap KR (1993) Acquisition of platinum drug resistance and platinum cross resistance patterns in a panel of human ovarian carcinoma xenografts. *Br J Cancer* **67**: 24–29

Jones NA, Turner J, McIlwrath AJ, Brown R and Dive C (1998) Cisplatin- and paclitaxel-induced apoptosis of ovarian carcinoma cells and the relationship between Bax and Bak up-regulation and the functional status of p53. *Mol Pharmacol* **53**: 819–826

Kelland LR, Mistry P, Abel G, Loh SY, O'Neill CF, Murrer BA and Harrap KR (1992*a*) Mechanism-related circumvention of acquired *cis*-diamminedichloro platinum(II) resistance using two pairs of human ovarian carcinoma cell lines by ammine/amine platinum (IV) dicarboxylates. *Cancer Res* **52**: 3857–3864

Kelland LR, Jones M, Abel G, Valenti M, Gwynne JJ and Harrap KR (1992*b*) Human ovarian carcinoma cell lines and companion xenografts: a disease oriented approach to new platinum anticancer drug development. *Canc Chemother Pharmacol* **30**: 43–50

Lock RB and Stribinskiene L (1996) Dual modes of death induced by etoposide in human epithelial tumor cells allow Bcl-2 to inhibit apoptosis without affecting clonogenic survival. *Cancer Res* **56**: 4006–4012

Ormerod MG, O'Neill C, Robertson D, Kelland LR and Harrap KR (1996) Cis-Diamminedichloro platinum (II)-induced cell death through apoptosis in sensitive and resistant human ovarian carcinoma cell lines. *Cancer Chemother Pharmacol* **37**: 463–471

Reed JC (1997) BCL-2 family proteins: role in dysregulation of apoptosis and chemoresistance in cancer. In: *Apoptosis and Cancer*, Martin SJ (ed), pp. 64–97. Karger-Landes Systems: Basel

Yin DX and Schimke RT (1995) *BCL-2* expression delays drug-induced apoptosis but does not increase clonogenic survival after drug treatment in HeLa cells. *Cancer Res* **55**: 4922–4928

*© 2000 Cancer Research Campaign*
